# Invasive plants as potential food resource for native pollinators: A case study with two invasive species and a generalist bumble bee

**DOI:** 10.1038/s41598-017-16054-5

**Published:** 2017-11-24

**Authors:** Maxime Drossart, Denis Michez, Maryse Vanderplanck

**Affiliations:** Laboratory of Zoology, Research Institute for Biosciences, University of Mons - UMONS, Place du Parc 20, B-7000 Mons, Belgium

## Abstract

It is now well established that invasive plants may induce drifts in the quantity and/or quality of floral resources. They are then often pointed out as a potential driver of bee decline. However, their impact on bee population remains quite unclear and still controversial, as bee responses are highly variable among species. Here, we compared the amino acid composition of pollen from three native and two invasive plant species included in diets of common pollinators in NW Europe. Moreover, the nutritional intake (i.e., pollen and amino acid intakes) of *Bombus terrestris* colonies and the pollen foraging behaviour of workers (i.e., visiting rate, number of foraging trips, weight of pollen loads) were considered. We found significant differences in pollen nutrients among the studied species according to the plant invasive behaviour. We also found significant differences in pollen foraging behaviour according to the plant species, from few to several foraging trips carrying small or large pollen loads. Such behavioural differences directly impacted the pollen intake but depended more likely on plant morphology rather than on plant invasive behaviour. These results suggest that common generalist bumble bees might not always suffer from plant invasions, depending on their behavioural plasticity and nutritional requirements.

## Introduction

Invasive plant species are frequently pinpointed as drivers of bee decline^1–3^. They impact composition and structure of plant communities, altering the suitability of invaded habitats^2^. However, bee responses (e.g., behaviour, health, abundance) to plant invasions are highly variable (i.e., negative, positive or neutral), making difficult both understanding and prediction of their impact on pollination networks and bee conservation^4^. On one hand, invasive plants can harm bee populations by modifying the diversity and/or the relative abundance of native plant species^2^ as some bee species are not able to forage or develop on alternative plants (including invasive species) because of behavioural (e.g., flower handling, host recognition) and/or physiological constraints (e.g., toxin occurrence, nutrient deficiency)^5–7^. On the other hand, invasive plant species can be integrated in bee diet^2,8^ (e.g., *Impatiens glandulifera* ^9–11^). Such new resource potential, coupled with massive, accessible and/or attractive floral display, probably explains local abundance and wide diversity of generalist bee species on some invasive plants^2,9^. However, foraging on a new host-plant (e.g., an invasive one that becomes predominant in the plant community) can also impact global diet quality^4^ and disrupt the cost/benefits balance of the foraging activity, depending on bee traits like physiological abilities (i.e., digestion) or sociality.

As mentioned by Heinrich^12^, a bumble bee colony can be sensed like an economic system in which energetic costs must not be higher than benefits. Its success is based on the optimization of energetic activities, with workers maximizing resource collection in both quantity^12^ and quality^13,14^ but spending the minimum of energy and time (e.g., in handling flower, flying or carrying loads)^12,15,16^. Changes in plant communities due to invaders could lead to predominance of new floral morphologies as well as differences in plant densities that pollinators have to deal with. Such modifications could then directly impact the energy balance of the foraging activity. Another key factor for the cost/benefit balance is probably the chemical composition of floral resources, including pollen^4^. Pollen provides several nutrients to bees (e.g., proteins, amino acids, lipids, carbohydrates, vitamins) but the concentration of these compounds is species-specific^17–19^. Among these nutritional compounds, total amino acids and proteins are known to play crucial role in bee metabolism, growth and development^20^. For this reason, they are often used as a proxy to assess pollen quality^21,22^. According to available data, crude protein content (i.e., evaluated from nitrogen content) of pollen varies from 2.5% to 61% (i.e., w/w dry pollen) while the total amino acid concentration ranges approximately from 20% to 50% (i.e., w/w dry pollen)^17,18^. According to these morphological and nutritive changes induced in plant communities, the reduction of plant diversity could then be detrimental for bees in the case of pollen nutrient decreases^23,24^.

Many studies have already addressed the attractiveness of invasive species for bees^5,25–28^, but only little attention has been paid to the direct impacts of plant invasions on the pollen foraging cost/benefit balance of bees. In particular, experimental studies that accurately evaluate invasive plant impact on nutritional intake of generalist species such as bumble bees are still lacking. In this work, we aimed (i) to compare the chemical composition of pollen from three native plant species, namely *Lythrum salicaria*, *Calluna vulgaris* and *Trifolium pratense*; as well as two invasive ones, namely *Buddleia davidii* and *Impatiens glandulifera*, (ii) to determine whether workers display the same pollen foraging pattern on these species regardless of their invasive behaviour, and (iii) to evaluate whether invasive species affect the nutritional intake of a very common European native generalist bumble bee species, *Bombus terrestris*. Our hypothesis is that the two invasive species display an easily accessible pollen resource for the buff-tailed bumble bee, with a similar nutritive composition to the three native ones.

## Results

### Host-plant quality

#### Floral pollen

The total amino acid content was significantly lower in the pollen from the two invasive species (F_1,13_ = 8.13, *P = *0.01). A significant difference was also detected among the different plant species (F_4,10_ = 7.55, *P = *0.004; Table 1) with the highest concentrations recorded in pollen from *L*. *salicaria* (315.23 mg/g dry matter) and *T*. *pratense* (403.39 mg/g dry matter). Although the relative proportion of essential amino acids (EAA) was not significantly different among the plant species (χ^2^ = 8.9, df = 4, *P = *0.063; Table 1), the concentration of EAA was significantly lower in the pollen from the two invasive species (F_1,13_ = 5.22, *P* = 0.04). Whereas no discrimination occurred according to plant invasive behaviour based on the aspartate and glutamate content of their pollen (i.e., nitrogen-rich amino acids implicated in universal metabolic processes) (F_1,13_ = 1.68, *P* = 0.22), a significant difference among plant species was detected (F_4,10_ = 5.32, *P = *0.015; Table 1). Actually the pollen from *L*. *salicaria*, *B*. *davidii* and *C*. *vulgaris* displayed the highest content in aspartate and glutamate (Table 1). The proline content of pollen differed according to the plant invasive behaviour (F_1,13_ = 21.63, *P < *0.001) as well as the plant species (F_4,10_ = 77.48, *P < *0.001). The post-hoc test arranged the different plant species into three groups: (i) one for *T*. *pratense* whose pollen displayed the highest proline content, (ii) one for *C*. *vulgaris* and *L*. *salicaria* whose pollen contained an intermediate proline content, and (iii) one for *B*. *davidii* and *I*. *glandulifera* whose pollen displayed the lowest proline content (Table 1

**Table 1:** The concentration (mg/g) of total and essential amino acids as well as the concentration of aspartate/glutamate and proline for the pollens of the five host-plants (mean ± sd). Values with the same letter are not significantly different.

Plant species	TAA content (mg/g)		EAA content (mg/g)		Asp and Glu content (mg/g)		Proline content (mg/g)	
*Buddleia davidii* (n = 3)	273.94 ± 19.81	**b**	135.53 ± 10.24	**a**	64.77 ± 3.83	**ab**	16.24 ± 1.04	**c**
*Calluna vulgaris* (n = 3)	290 ± 2.52	**b**	145.73 ± 1.06	**a**	62.04 ± 1	**ab**	22.9 ± 0.3	**b**
*Impatiens glandulifera* (n = 3)	252.85 ± 52.91	**b**	134.05 ± 28.56	**a**	54.12 ± 10.18	**b**	11.18 ± 2.9	**c**
*Lythrum salicaria* (n = 3)	315.23 ± 10.53	**ab**	150.8 ± 4.63	**a**	76.3 ± 3.07	**a**	27.65 ± 2.37	**b**
*Trifolium pratense* (n = 3)	403.39 ± 29.47	**a**	288.27 ± 20.6	**a**	58.72 ± 8.2	**b**	40.82 ± 3.17	**a**
Statistical results	F_4,10_ = 7.55, *P* = 0.004		H = 8.9, df = 4, *P* = 0.063		F_4,10_ = 5.32, *P* = 0.015		F_4,10_ = 77.48, *P* = 0.001	

).

**Figure 1:**
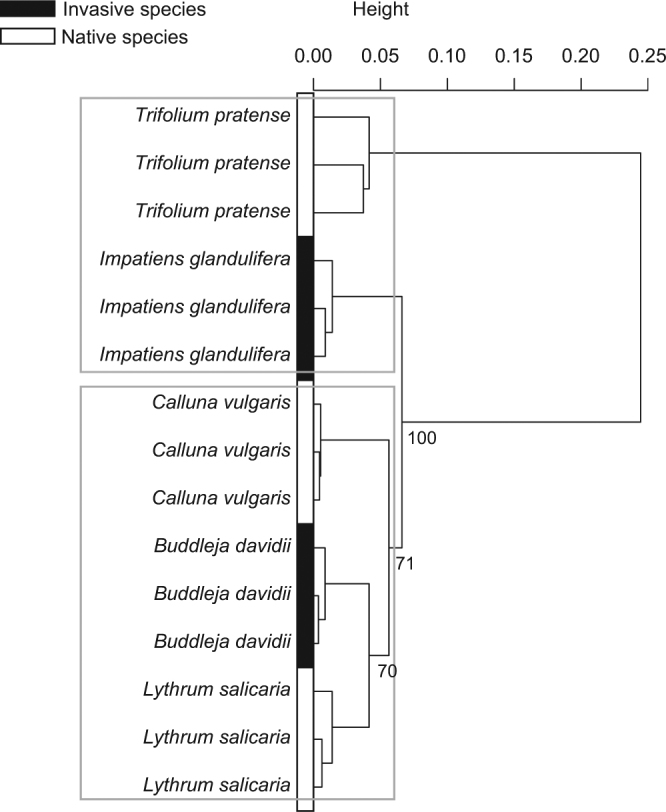
Pollen chemistry. Cluster based on Bray-Curtis dissimilarity index calculated on the relative proportions (%) of total amino acids in pollen from the five host-plant species (n = 3 for all studied plant species).

Although the plant species were significantly different from each other according to their pollen amino acid profile (F_4,10_ = 192.96, *P = *0.001), the perMANOVA did not detect any significant difference according to the invasive behaviour. Pairwise comparisons arranged the different host-plants into two groups: (i) one with *T*. *pratense* and *I*. *glandulifera*, and (ii) one with *C*. *vulgaris*, *B*. *davidii* and *L*. *salicaria*. Although native and invasive species were mixed in the cluster (Fig. 1), indicator compound analysis showed that histidine was significantly associated with the two species displaying an invasive behaviour, namely *B*. *davidii* and *I*. *glandulifera* (*P = *0.017, indicator value = 0.56; Table S1), and proline with the native ones, namely *T*. *pratense*, *C*. *vulgaris* and *L*. *salicaria* (*P = *0.048, indicator value = 0.63; Table S1).

#### Pollen loads

Pollen loads from workers when foraging on the invasive plants (i.e., *B*. *davidii* and *I*. *glandulifera*) were significantly less concentrated in total amino acids compared to those when foraging on the native species (F_1,12_ = 5.81, *P = *0.032; Table 2). Significant difference was also detected according to the botanical origin of the pollen loads (F_4,9_ = 6.46, *P = *0.01; Table 2), with the highest concentrations recorded in loads coming from *C*. *vulgaris* and *T*. *pratense* (193.33 and 184.5 mg/g dry matter, respectively). With regards to essential amino acid and aspartate/glutamate contents of pollen loads, no significant difference was detected among plant species or according to their invasive behaviour (*P* > 0.05; Table 2). By contrast, pollen loads from the studied invasive plant species (i.e., *B*. *davidii* and *I*. *glandulifera*) displayed a significantly lower content in proline compared to those from native plant species investigated herein (F_1,12_ = 60.75, *P < *0.001). A difference was also detected among the plant species that were arranged in four groups (F_4,9_ = 125.7, *P < *0.001; Table 2): (i) one for *T*. *pratense*, (ii) one for *C*. *vulgaris*, (iii) one for *L*. *salicaria*, and (iii) one for both *B*. *davidii* and *I*. *glandulifera* (Table 2

**Table 2:** The concentration of total and essential amino acids as well as the concentration of aspartate/glutamate and proline for the pollen loads coming from the five host-plants (mean ± sd). Values with the same letter are not significantly different.

Plant species	TAA content (mg/g)		EAA content (mg/g)		Asp and Glu content (mg/g)		Proline content (mg/g)	
*Buddleia davidii* (n = 3)	151.72 ± 16.44	**b**	78.71 ± 8.2	**a**	37.81 ± 4.1	**a**	7.73 ± 1.18	**d**
*Calluna vulgaris* (n = 3)	193.33 ± 1.45	**a**	94.96 ± 0.34	**a**	39.87 ± 0.35	**a**	20.11 ± 0.79	**b**
*Impatiens glandulifera* (n = 3)	149.31 ± 6.71	**b**	76.61 ± 3.38	**a**	35.8 ± 1.25	**a**	6.24 ± 0.1	**d**
*Lythrum salicaria* (n = 2)	144.03 ± 5.4	**b**	70 ± 3.14	**a**	33.77 ± 0.51	**a**	13.94 ± 0.86	**c**
*Trifolium pratense* (n = 3)	184.5 ± 26.6	**ab**	84.63 ± 13.48	**a**	37.8 ± 4.56	**a**	28.97 ± 3.72	**a**
Statistical results	F_4,9_ = 6.46, *P* = 0.01		H = 6.8, df = 4, *P*= 0.15		H = 5.95 df= 4, *P*= 0.2		F*4,9* = 125.7 *P* < 0.001	

). The former displayed the highest proline content and the latter the lowest one.

**Figure 2:**
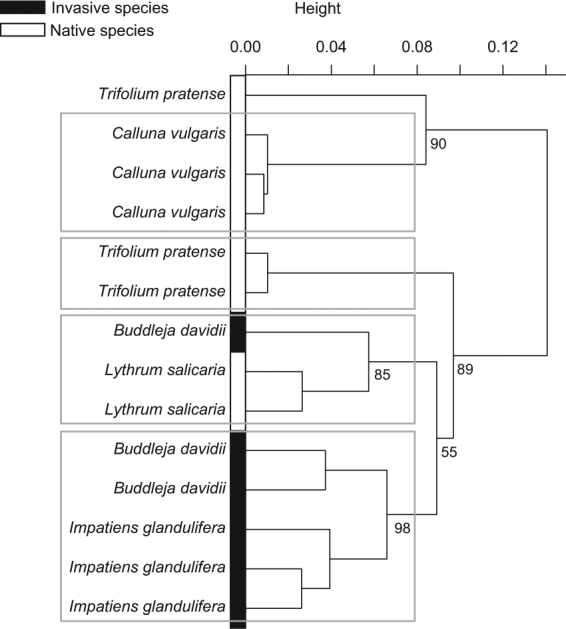
Pollen loads chemistry. Cluster based on Bray-Curtis dissimilarity index calculated on the relative proportions (%) of total amino acids in pollen loads from the five host-plant species (n = 3 for all studied plant species, except *L*. *salicaria* n = 2).

With regards to amino acid profile, interspecific perMANOVA (F_4,9_ = 8.18, *P = *0.001) and pairwise comparisons showed that pollen loads from the three native plants differed from each other (*P* < 0.05; Fig. 2) whereas those from the investigated species displaying an invasive behaviour (i.e., *B*. *davidii* and *I*. *glandulifera*) were not significantly different (F_1,4_ = 0.29, *P* = 0.221; Fig. 2). Although *B*. *davidii* pollen did not significantly differ from *L*. *salicaria* pollen (F_1,3_ = 0.43, *P* = 0.21; Fig. 1), perMANOVA conducted on plant invasive behaviour revealed that pollen from the studied invasive species significantly differed from pollen from the native ones herein investigated (F_1,12_ = 5.09, *P = *0.043). According to indicator compound analysis, pollen loads from native plant species were significantly associated with proline (*P = *0.017, indicator value = 0.76; Table S2).

### Foraging parameters

#### Visiting rate

**Figure 3:**
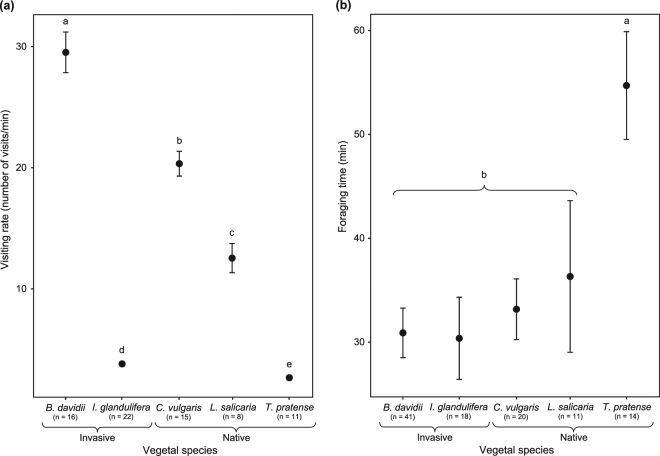
Foraging behaviour. Visiting rate (**a**) and foraging time (**b**) according to the plant species. Species with the same letter are not significantly different.

Visiting rate was plant species-dependent (F_4,46.19_ = 192.54, *P < *0.001), which means that bumble bee workers visited a variable number of flowers in a given time interval depending on the displayed plant species. Post-hoc analysis did not allow defining groups (Fig. 3a). With regards to invasive behaviour, there was no significant difference between native and invasive species herein investigated (F_1,66.29_ = 1.5, *P = *0.22). Actually *B*. *davidii* displayed the highest visiting rate (29.53 ± 6.71 per floral unit) and *I*. *glandulifera* the lowest one (3.81 ± 0.81 per floral unit) (Fig. 3a; Table S3).

#### Foraging time and number of foraging trips

By contrast to the visiting rate, duration of foraging trip did not depend on the plant species (i.e., around 32 ± 16 min; pairwise comparisons, *P* > 0.05) except for *T*. *pratense* (i.e., around 55 ± 19 min; ANOVA, F_4,66.11_ = 4.13, *P = *0.0047; Table S3) (Fig. 3b). Two groups were defined by post-hoc analysis: (i) one for *T*. *pratense* and (ii) one for the other plant species (Fig. 3b). Foraging time was not related to invasive behaviour as no significant difference was detected between the two native and the three invasive species (F_1,96.35_ = 3.44, *P = *0.067).

The number of foraging trips in a given interval differed significantly among the plant species (χ^2^ = 25.36, df = 4, *P < *0.001; Table 3). According to the pairwise comparisons, the workers performed significantly more foraging trips in presence of *B*. *davidii* compared to the other plant species. However, the plant invasive behaviour did not impact the number of worker trips back to the colony with pollen loads (Table 3

**Table 3:** The number of bouts performed by workers during 16 h as well as the mean weight of pollen loads brought back during this time period for the five host-plants. Values with the same letter are not significantly different.

Plant species	Number of foraging trips		Weight of pollen loads (mg)	
*Trifolium pratense*	15	**b**	10.28 ± 2.82	**a**
*Lythrum salicaria*	12	**b**	5.73 ± 1.03	**b**
*Calluna vulgaris*	22	**b**	3.24 ± 1.67	**d**
*Buddleia davidii*	42	**a**	3.67 ± 1.19	**c**
*Impatiens glandulifera*	19	**b**	4.47 ± 1.82	**bc**
Statistical results	χ^2^ = 25.36, df = 4, *P* < 0.001		F_4,57.68_ = 22.16, *P* < 0.001	

).

#### Nutritional intake

The weights of pollen loads brought back to the colonies were significantly different among species (F_4,57.68_ = 22.16, *P < *0.001; Table 3). Detailed analysis revealed that workers made heavier pollen loads when foraging on *T*. *pratense* (10.28 ± 2.82 mg) compared to the other plant species (P < 0.05). Moreover, weight of pollen loads appeared quite variable among the native plant species as workers foraging on *T*. *pratense* displayed the heaviest pollen loads and those foraging on *C*. *vulgaris* the lightest ones (Table 3). By contrast, pollen loads from workers foraging on invasive plants were quite similar with an intermediate weight compared to those made of native pollen (Table 3). As expected, the weight of pollen load did not significantly differ between the investigated native and invasive species (F_1,107.94_ = 1.34, *P* = 0.25).

**Figure 4:**
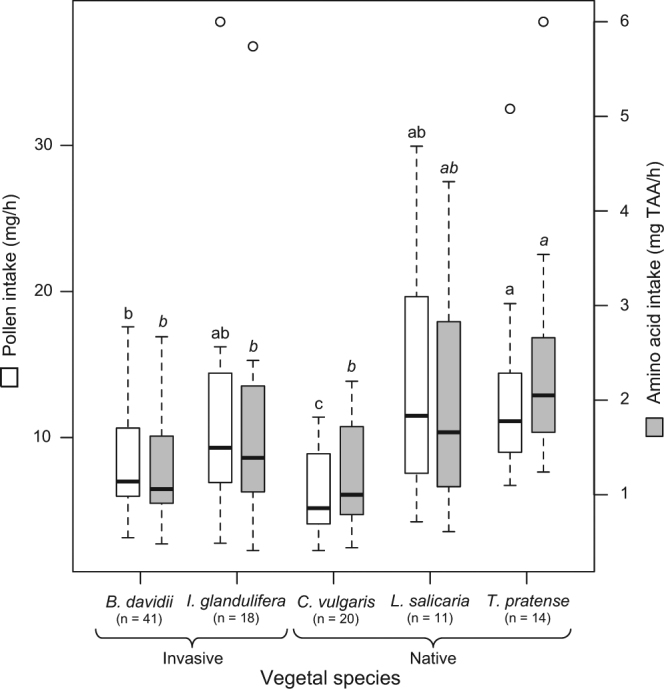
Foraging efficacy. Expressed as pollen intake (i.e., mg/h, in white) and nutritive intake (i.e., TAA mg/h, in grey) according to the visited plant species. Species with the same letter are not significantly different.

Considering both the mass of pollen loads and the number of foraging trips in a given interval (i.e., pollen efficacy, mg/h), analyses showed that workers brought significantly less pollen back to the colony when foraging on *C*. *vulgaris* (6.29 ± 2.86 mg/h) compared to the other plant species (F_4,44.94_ = 4.64, *P = *0.003; Fig. 4; Table S3). The highest pollen intake was reached when workers foraged on *T*. *pratense* (13.12 ± 6.6 mg/h) whereas pollen intake was intermediate when workers foraged on *B*. *davidii*, *I*. *glandulifera* and *L*. *salicaria* (Fig. 4; Table S3). As expected with regards to these interspecific comparisons, no significant difference was detected according to the plant invasive behaviour (F_1,101.41_ = 0.62, *P = *0.43; Fig. 4).

Considering both pollen intake and quality of pollen loads (i.e., nutritive efficacy, mg TAA/h), interspecific comparisons revealed that amino acid intake was higher when workers foraged on *T*. *pratense* (2.42 ± 1.22 mg TAA/h) compared to *B*. *davidii* (1.29 ± 0.58 mg TAA/h), *C*. *vulgaris* (1.22 ± 0.55 mg TAA/h) and *I*. *glandulifera* (1.68 ± 1.2 mg TAA/h) (F_4,43.16_ = 3.77, *P = *0.01; Fig. 4; Table S3). Amino acid intake when foraging on *L*. *salicaria* was intermediate (Fig. 4). No significant difference was detected between native and invasive species (F_1,100.02_ = 0.96, *P* = 0.33; Fig. 4).

## Discussion

### Chemical suitability of *Buddleia davidii* and *Impatiens glandulifera*

In term of chemical composition, floral pollen and pollen loads showed variable amino acid concentrations and compositions according to their botanical origin, which is in congruence with previous studies^8,29,30^. Despite this variability, pollen from the five plant species contained the full spectrum of essential amino acids (i.e., no amino acid deficiency). Such consistency of the full spectrum in pollen has been already mentioned by Weiner *et al*.^29^ as well as Roger *et al*.^30^ based on a larger sampling of plants. In a diverse vegetal community, some invasive plants can obviously provide suitable pollen resource for bumble bees considering their amino acid composition of their pollen.

While chemical composition of pollen is probably related to the attractiveness to pollinators^14^, it has also an important role in the health and fitness of bees^22,31,32^. Especially, chemical composition of pollen loads brought back to the nest by bumble bee workers may impact whole colony development^33^. Our results revealed that pollen loads from the studied invasive plants (i.e., *B*. *davidii* and *I*. *glandulifera*) had on average lower concentrations of proline compared to those from native species. However, caution has to be paid since the proline content was highly variable among the native plants investigated. Although being non-essential amino acid, proline is highly important for bumble bees as it is involved in the flight metabolism^34,35^. Low proline concentration in a pollen diet could then directly impact the floral attractiveness as well as the foraging efficiency of workers and consequently the cost/benefit balance of the colony. By contrast, histidine was more abundant in pollen of *Impatiens glandulifera*, than in pollen of native ones as already shown by Harmon-Threatt and Kremen^4^. This chemical pollen trait might ensure the integration of *I*. *glandulifera* in generalist bumble bee diet (i.e., attractiveness to pollinators^36^) as well as the availability of the essential histidine in case of lack in some plant communities^4^.

Overall, despite these differences in the relative abundances of some amino acids in their pollen, *I*. *glandulifera* and *B*. *davidii* provide resources not consistently different in terms of amino acids from native plants, suggesting that generalist bumble bees may use them without change in their global pollen diet^4,30^.

### Foraging behaviour and nutritional intake

According to our results, different pollen foraging behaviours may be described for the five plant species: (i) a few foraging trips carrying small pollen loads (e.g., workers foraging on *C*. *vulgaris* and to a lesser extent on *I*. *glandulifera*), (ii) many foraging trips carrying small pollen loads (e.g., workers foraging on *B*. *davidii*), and (iii) a few foraging trips carrying large pollen loads (e.g., workers foraging on *T*. *pratense* and to lesser extent on *L*. *salicaria*). All these pollen foraging behaviours are likely related to the floral morphology of the host-plants. Actually it is commonly accepted that bee pollen foraging behaviour is highly dependent on floral symmetry of the host-plant, radial flowers being usually easier to handle than zygomorphic ones^37^. Variation in floral morphology of the selected plant species, regardless of plant invasive behaviour, is then expected to impact the different pollen foraging parameters herein considered, such as the visiting rate. Actually, floral display of *Buddleia davidii* (i.e., dense inflorescence) and to a lesser extent of *C*. *vulgaris* and *L*. *salicaria* allows the workers to forage on a high number of flowers in a given interval. These results are in line with other studies that have already suggested that flowers of *C*. *vulgaris* arranged in dense bunches as well as the plant morphology organized in bush allow workers walking from flower to flower, optimizing their visiting rate^12^. Such observations are likely true for *T*. *pratense*, although data were not comparable because of the different count unit due to spherical and dense inflorescence of red clover (i.e., inflorescence unit for *T*. *pratense* and floral unit for other plant species). Nevertheless, hypothesis of high foraging efficiency on *T*. *pratense* is corroborated by previous studies showing that, in spite of their complex morphology (i.e., zygomorphic flowers), Fabaceae flowers are easily foraged by buff-tailed bumble bees and constitute valuable host-plants^12,38–40^. By contrast to the aforementioned plant species, the visiting rate for *I*. *glandulifera* is low, suggesting that workers require more time to handle each flower. This observation could be partly explained by the slender structure of this species, as it likely involves a higher energetic and temporal investment (i.e., flying from flower to flower) than for dense inflorescence. Alternative hypothesis is that floral dimensions could announce an abundance of resources, inciting workers to spend more time per flower of *I*. *glandulifera* ^37,41^.

Considering both pollen foraging behaviour and pollen chemical composition, foraging on *T*. *pratense* provided the highest nutritive intake. Evidence is that the nutritional intake was related to both host-plant morphology and pollen quality rather than to the host-plant invasive behaviour. However, further studies are still needed to corroborate this finding, taking into account invasive plants with peculiar (i.e., unusual) flower morphologies and pollen composition.

### Invasive plants and bee decline

The spread of invasive plant species directly impacts the plant community composition of invaded sites, leading to losses in plant diversity^2,5,42,43^. Such decrease in plant diversity would be detrimental to pollinator health by affecting the nutritional intake of bees^4,23^. It could then cause bumble bee species decline in both diversity and abundance as observed during the last decades (e.g., *Bombus jonellus*, *B*. *soroeensis*)^44–48^. However, some bumble bee species have better or neutral responses to the ongoing global change, including changes in plant diversity in highly disturbed areas (e.g., *B*. *pascuorum*, *B*. *terrestris*)^49–51^. According to previous studies, these stable and expanding bumble bee species are able to maintain their nutritional intake while incorporating new pollen resources in their diet, including invasive plant species^4,30,50^. Actually, our results coupled with previous studies^4,30^, revealed that no main difference seems occurred according to the plant invasive behaviour with regard to amino acid content of pollen, even if proline was relatively more abundant in pollen of the three native plant species and histidine in pollen of *I*. *glandulifera*. In the same way, pollen and nutritional intakes of the colonies did not depend on the plant invasive behaviour but were highly variable among the five studied species. Our results suggest that predominance of new floral morphologies in a plant community following a plant invasion could more likely disrupt the cost/benefit balance of bumble bee colonies than any change in amino acid availability. *I*. *glandulifera* as well as *B*. *davidii* could be valuable pollen resources for *B*. *terrestris* despite their invasive behaviour. Removing beneficial plants like *I*. *glandulifera* and *B*. *davidii*, representing major food sources, could then be detrimental for generalist bees in forage-depleted agri-environments^2,52^ and then for plant communities as well^53^.

However incorporation of such invasive species in pollination networks impacts ecosystem more than just suitability for generalist bumble bees able to face different floral morphologies (i.e., *B*. *terrestris*)^4,30,50^. Actually, some bumble bee species display a lower plasticity in their pollen diet (i.e., new host incorporation) such as the long-tongued *B*. *hortorum* morphologically linked to the red clover *T*. *pratense* ^30,54^. In the same way, native bumble bee species specialized on peculiar native plant species (e.g., *B*. *jonellus* on Ericaceae) could be affected by the spread of invasive species^2,50^ since they could compete with native plants for nutrients, water, light, space and reproduction^9,55^. This could also impact a large array of oligolectic bees that are intimately linked to native plants (e.g., *B*. *jonellus* on *Vaccinium* species, *Melitta nigricans* (Melittidae) and *Tetraloniella salicariae* (Apidae) on *L*. *salicaria*) could then be affected^2,50,56^ and sometimes not able to develop on non-host pollens^6^.

Given the great bee diversity, further studies are needed on generalist species as well as specialists for a better understanding of the impact of invaders. Such understanding is clearly necessary to develop mitigation strategies for maintaining the bee diversity as well as the inherent ecosystem services.

## Material and Methods

### Biological models

**Figure 5:**
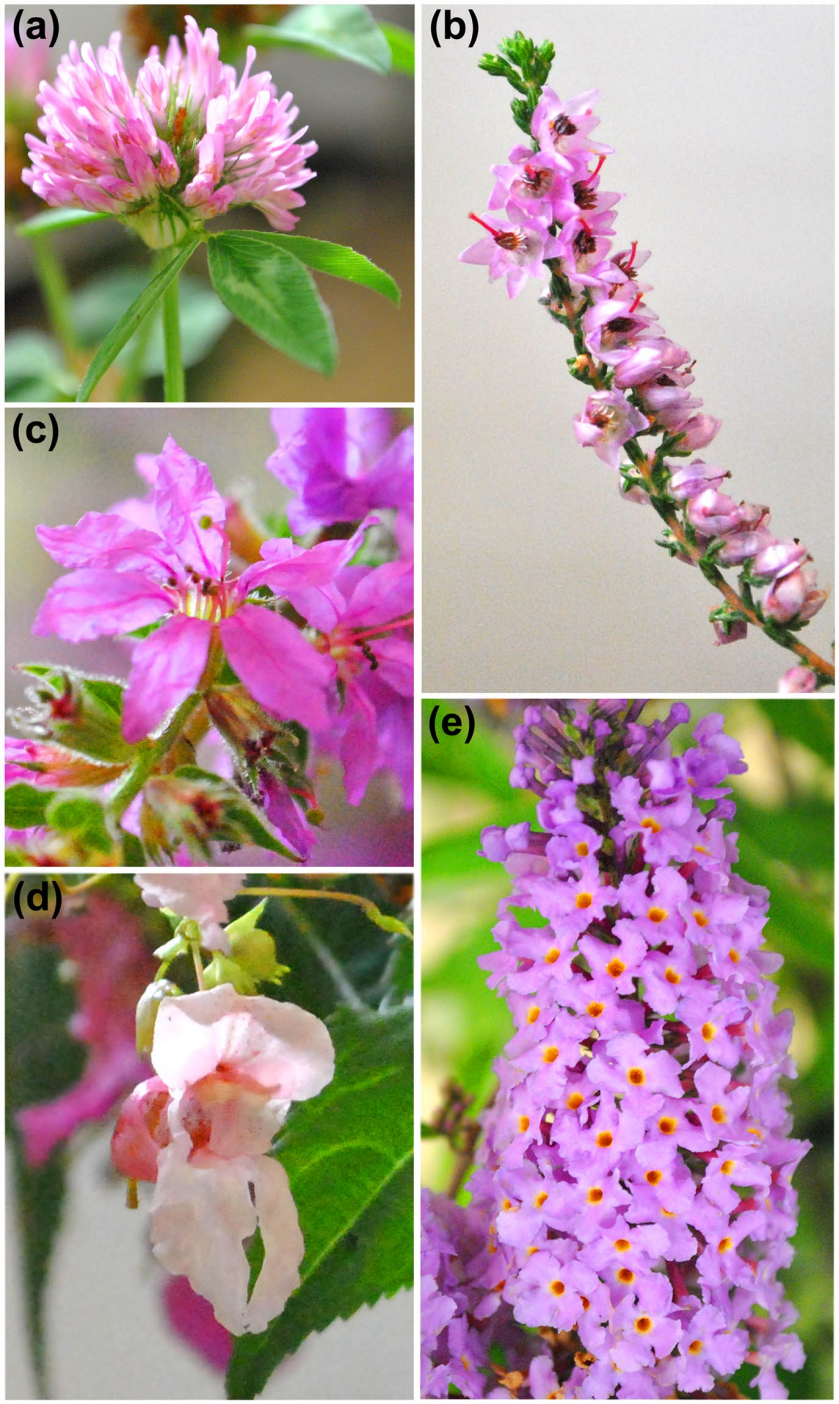
Studied plant species. Photographs of plant species with (**a**) *Trifolium pratense*, (**b**) C*alluna vulgaris*, (**c**) *Lythrum salicaria*, (**d**) *Impatiens glandulifera* and (**d**) *Buddleia davidii*.

*Bombus terrestris* was selected as model for generalist bee species because colonies are commercialized, easy to rear and this species includes pollen from invasive species in its diet^50,57^. Moreover, it is one of the most abundant bumble bee species in the West-Palaearctic region^58^. *Trifolium pratense*, *Calluna vulgaris* and *Lythrum salicaria* were selected as important native host-plant of bumble bees^50^. Among the numerous invasive plants in NW Europe^9,43^ we selected two species that were integrated in the pollen diet of numerous bees, including *Bombus terrestris* ^50^: *Impatiens glandulifera* and *Buddleia davidii*. These two species can be very aggressive in their invasion process^5,59^ and some countries in Europe are developing plans against these invasive plants^60,61^. They can be temporally the unique food resources for bees in invaded areas^43^. The five plant models are blooming in the same time in NW Europe. They display a distinct floral morphology: while *T*. *pratense* and *I*. *glandulifera* show small and large zygomorphic flowers, respectively; the floral symmetry of other species is radial with various petal morphologies^37,62^ (Fig. 5).

### Host-plant quality

#### Chemical composition of floral pollen

Total amino acid (i.e., free and protein-bound) concentrations and profiles (i.e., relative proportions of each amino acid) have been assessed for each plant, with some of these data being already available from literature^30^. For each species, pollen grains were removed from fresh anthers with a tuning fork and gathered on a glass plate. These samples were lyophilized during 24 h and stored at −20 °C before chemical analyses. Amino acid compositions were assessed in triplicate of 3–5 mg of floral pollen (dry weight) following the protocol explained in Vanderplanck *et al*.^22^. Total amino acids were measured separately with an ion exchange chromatograph (Biochrom 20 plus amino acid analyzer) using norleucine as internal standard. Only tryptophan was omitted because its isolation requires a separate alkaline hydrolysis from additional amounts of sample, and it is hardly ever a limiting essential amino acid^63^.

#### Chemical composition of pollen loads

**Figure 6:**
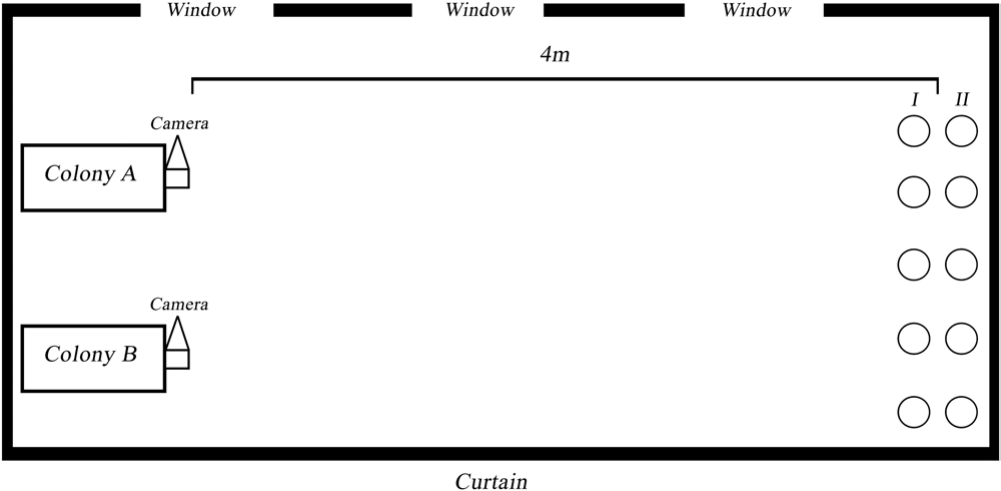
Experimental diagram. Cameras were paired with entrance of colonies in order to record arrivals and exits of workers. Plants were placed in two rows: “I” referred to plants placed on the floor and “II” to plants placed at height.

As bees can add salivary enzymes, nectar and microorganisms to the pollen stored in their corbiculae (e.g., bumble bees and honey bees)^22,64–66^, we also considered the chemical composition of pollen loads in a second group of analyses. Two colonies of *B*. *terrestris* provided by Biobest *bvba* (Waterlo, Belgium) were placed in an enclosed area at constant temperature and relative humidity (23 ± 1 °C and 50%, respectively). An Everglades air-conditioner (EV 9057 1050 W) as well as two AEG wall convection heaters (WKL 2003 U 2 kW) enabled to keep a constant ambient temperature. Plant bouquets were collected in wild populations during September 2013 (Mons, Belgium). Each plant species was randomly and separately provided to colonies during two successive days (i.e., 48 h) (*I*. *glandulifera*, *T*. *pratense*, *L*. *salicaria*, *C*. *vulgaris* and *B*. *davidii*, respectively). Such experimental design implied that colonies were constantly faced to a single food source at a time and then forced to forage on. This design cancelled the bias due to floral preference and allowed to quantify nutritional intake for all the plant species investigated. Regarding the number of available flowers for the different plant species, we provided sets of floral bouquets with a similar floral density to the colonies. These sets of floral bouquets were put in two rows at the other side of the room (i.e., I and II at 4 m from colonies; Fig. 6).

At the end of their foraging trips, workers were immobilized in a “turn and mark” tube to remove from their corbiculae one of both pollen loads (i.e., to avoid them stopping foraging^67^). We collected pollen loads to obtain a final mass around 5–15 mg for amino acid analyses. These chemical analyses have been performed following the same method described above.

### Pollen foraging parameters

As highlighted by Raine & Chittka^67^, handle flowers for collecting pollen might be complex (i.e., learning to handle, collect and pack pollen for transport back to the nest). Pollen collection is then often more energetically costly than nectar one. Whereas nectar reward impacts more likely plant attractiveness and bee floral choices, pollen accessibility directly impacts foraging behaviour (e.g., foraging time, visitation rate) and then the cost/benefit balance of the foraging activity.

As the aforementioned experimental design did not consider floral choices by bees (i.e., each plant was separately presented to colonies), nectar was not a valuable parameter for testing our hypothesis and only pollen foraging was herein considered^67–70^. However, nectar should be considered in studies investigating floral preferences and invasive plant fitness.

For monitoring workers, all the individuals of the two colonies were marked with unique numbered and coloured tags. The entries of colonies were connected to airlocks constituted by a transparent tube in order to slow down the workers. Cameras (Panasonic HC-V100 High Definition) were placed in front of these airlocks to record each arrival and exit of marked individuals. The total recording time for each plant was 18 h (i.e., 2 cycles of 9 hours of observation). We considered five parameters: (i) visiting rate; (ii) visiting time; (iii) foraging time; (iv) number of foraging trips; and (v) nutritional intake.

#### Visiting rate and visiting time

Each hour during the recording period (i.e., from 8 am until 5 pm), several individuals were followed-up and timed with a chronometer during their foraging trip in order to evaluate the number and the duration of each floral visit during 15 minutes. Floral visit time was considered from the first flower contact till the last one^71^. The visiting rate was defined as the number of floral visits per minute, for each forager on each plant species. It has to be noticed that the visiting rate for *T*. *pratense* was so high (i.e., flowers grouped in globular inflorescence) that we had to consider the inflorescence rather than the flower as count unit.

#### Foraging time and number of foraging trips

The foraging time corresponded to the time between the exit and the return of one worker into the colony. Films were analysed at different reading speeds (i.e., *1-*2-*4-*8) using the Windows Live Movie Maker program. Each back and forth has then been recorded in order to determine the duration of each foraging trip. The number of foraging trips was defined as the number of worker returns to the colonies. This parameter was determined for each plant species during all the recording time (i.e., 2*9 hours) based on same films than for the foraging time parameter.

#### Nutritional intake

The nutritional intake was defined as the pollen and amino acid intakes per hour. In order to determine the weight of each pollen load brought back to the colony, a relationship between the weight and the area of pollen loads has been established for each plant species using sampling sets (i.e., pollen loads removed from workers with measure of their surface and weight). The areas of pollen loads were determined using Nikon D3000 camera and ImageJ ® software. The relation between surface and weight of pollen load was determined for each plant species based on a simple linear regression (R-package “lmtest”) after checking for autocorrelation (Durbin-Watson test), homoscedasticity (Breush-Pagan test) and normality of residuals (Shapiro test). The relations were defined as y = 0.126x (R² = 0.64, *P* = 0.006) for *I*. *glandulifera*, y = 0.206x (R² = 0.78, *P* = 0.029) for *T*. *pratense*, y = 0.122x (R² = 0.93, *P* = 0.005) for *L*. *salicaria*, y = 104x (R² = 0.97, *P* = 0.01) for *C*. *vulgaris* and y = 0.127x (R² = 0.87, *P* < 0.001) for *B*. *davidii*. The weights of pollen loads brought back to the colonies were inferred using 2D pictures obtained from movies (i.e., each back of workers has been recorded) and aforementioned linear relation. The total pollen income per hour per colony (mg/h) as well as the total amino acid income per hour per colony (mg/h) were then determined using data from foraging time and pollen load analyses.

### Statistical analysis

All data visualizations and analyses were conducted in R 3.0.2.

#### Floral pollen and pollen load composition

Two-way analyses of variance (Two-Way ANOVA) were performed on total amino acid content of floral pollen and pollen loads to compare native and invasive plants including plant species as nested factor. Since it is a parametric test, homoscedasticity (Bartlett test) and normality of the residuals (Shapiro test) were checked prior to the analyses. When violation occurred, data were log- or rank-transformed (“rntransform” command, R-package GenABEL). Multiple pairwise comparisons (i.e., post-hoc tests) were conducted when ANOVA detected significant difference among host-plants (*P < *0.05).

To compare total amino acid profile (i.e., relative abundances) of floral pollen and pollen loads from the different host-plants, permutational multivariate analyses of variance (perMANOVAs) and multiple pairwise comparisons (Bonferroni’s adjustment) were conducted using the Bray-Curtis dissimilarity index (calculated by R software during the analysis) and 999 permutations (“adonis” command, R-package vegan^72^). Prior to perMANOVA, the multivariate homogeneity of within-group covariance matrices was verified using the “betadisper” function implementing Marti Anderson’s testing methods. Differences in amino acid profiles were visually assessed on UPGMA clusters using the Bray-Curtis dissimilarity index. We assessed the uncertainty in hierarchical cluster analysis with p-values calculated via multiscale bootstrap resampling (R-package pvclust^73^). Indicator compound analyses were also performed in using the “indval” function from the labdsv package^74^ to identify the amino acids that were indicative of one host-plant (i.e., floral pollen or pollen loads). These multivariate analyses were performed using either plant invasive behaviour (i.e., invasive or native) or plant species as qualitative variable.

#### Foraging parameters

Numbers of foraging trips were compared using Pearson’s Chi-squared test for count data and either plant invasive behaviour or plant species as qualitative variable. Generalized linear mixed models (GLMMs) were used to analyse the effect of the plant invasive behaviour (i.e., invasive or native) and plant species (i.e., *B*. *davidii*, *C*. *vulgaris*, *I*. *glandulifera*, *L*. *salicaria* and *T*. *pratense*) on either visiting rate, foraging time or foraging efficacy expressed as pollen intake and amino acid intake, including the worker identification as random factor (“lmer” function, R-package “lmerTest”^75^). Normality of the residuals and data overdispersion were checked (*P* > 0.05). Data were log- or rank-transformed (“rntransform” command, R-package GenABEL) when violation occurred. Effects of fixed and random factors (i.e., ANOVA and difference of least squares means) were assessed using the “step” function implemented in the R-package “stats”^76^. Data were visually assessed on boxplots or plots of means according to the data distribution.

## Electronic supplementary material

Supplementary informations
