# Supplementary material for: Invasive plants as potential food resource for native pollinators: A case study with two invasive species and a generalist bumble bee
Source: Sci Rep. 2017 Nov 24;7:16242. doi: 10.1038/s41598-017-16054-5 (PMC5701216; doi:10.1038/s41598-017-16054-5)
Supplement: Supplementary file 1 — Supplementary informations [file 41598_2017_16054_MOESM1_ESM.doc]

**– Supplementary Information –**

**Invasive plants as potential food resource for native pollinators: A case study with two invasive species and a generalist bumble bee**

Maxime Drossart^1,*^, Denis Michez^1, **^ and Maryse Vanderplanck^1, **^

^1^ Laboratory of Zoology, Research Institute for Biosciences, University of Mons - UMONS, Place du Parc 20, B-7000 Mons, Belgium.

^*^ Corresponding author; +32 67 37 34 36; maxime.drossart@umons.ac.be

^**^ These authors co-supervised equally the present study

**Supplementary Table S1.** Amino acid composition from the floral pollens of invasive plants as well as native plants. The concentrations are expressed in mg/g (mean ± sd) and as percentage of total amino acids in brackets.

|  |  |  | **Invasive plants** | | | | |  | **Native plants** | | | | | | | |
| --- | --- | --- | --- | --- | --- | --- | --- | --- | --- | --- | --- | --- | --- | --- | --- | --- |
|  |  |  | *Impatiens glandulifera* (n=3) | |  | *Buddleia davidii* (n=3) | |  | *Calluna vulgaris* (n=3) | |  | *Lythrum salicaria* (n=3) | |  | *Trifolium pratense* (n=3) | |
|  |  |  |  |  |  |  |  |  |  |  |  |  |  |  |  |  |
| Essential amino acids | Arginine |  | 15.16 ± 3.45 | (6.00) |  | 19.28 ± 1.78 | (7.04) |  | 20.87 ± 0.17 | (7.20) |  | 20.74 ± 0.52 | (6.58) |  | 20.77 ± 1.15 | (5.15) |
|  | Histidine |  | 13.3 ± 2.84 | (5.25) |  | 10.31 ± 0.84 | (3.76) |  | 10.61 ± 0.08 | (3.66) |  | 11.71 ± 0.43 | (3.71) |  | 12.35 ± 0.89 | (3.06) |
|  | Isoleucine |  | 13.49 ± 2.98 | (5.34) |  | 14.79 ± 1.16 | (5.40) |  | 13.85 ± 0.05 | (4.78) |  | 15.19 ± 0.54 | (4.82) |  | 4.74 ± 3.07 | (1.17) |
|  | Leucine |  | 20.38 ± 4.03 | (8.06) |  | 22.31 ± 1.52 | (8.15) |  | 22.4 ± 0.18 | (7.73) |  | 24.9 ± 0.65 | (7.90) |  | 17.83 ± 1.02 | (4.42) |
|  | Lysine |  | 20.82 ± 4.18 | (8.24) |  | 22.32 ± 1.39 | (8.15) |  | 22.96 ± 0.44 | (7.92) |  | 25.32 ± 1.01 | (8.03) |  | 30.65 ± 2.54 | (7.60) |
|  | Methionine |  | 7.81 ± 2.04 | (3.09) |  | 0 | (0.00) |  | 8.57 ± 0.18 | (2.95) |  | 1.57 ± 0.26 | (0.50) |  | 21.64 ± 0.78 | (5.37) |
|  | Phenylalanine |  | 14.10 ± 3.34 | (5.57) |  | 15.93 ± 1.41 | (5.81) |  | 14.69 ± 0.08 | (5.07) |  | 16.32 ± 0.45 | (5.18) |  | 13.19 ± 1.14 | (3.27) |
|  | Threonine |  | 14.21 ± 2.94 | (5.62) |  | 14.59 ± 1.03 | (5.33) |  | 14.18 ± 0.11 | (4.89) |  | 16.48 ± 0.35 | (5.23) |  | 37.46 ± 3.84 | (9.29) |
|  | Valine |  | 14.81 ± 2.79 | (5.86) |  | 16 ± 1.1 | (5.84) |  | 17.59 ± 0.26 | (6.07) |  | 18.55 ± 0.74 | (5.89) |  | 21.84 ± 2.24 | (5.41) |
|  |  |  |  |  |  |  |  |  |  |  |  |  |  |  |  |  |
|  | Alanine |  | 15.77 ± 3.34 | (6.24) |  | 15.48 ± 1.12 | (5.65) |  | 14.7 ± 0.09 | (5.07) |  | 16.8 ± 0.56 | (5.33) |  | 15.34 ± 1.04 | (3.80) |
|  | **Aspartic acid** |  | 24.46 ± 4.6 | (9.68) |  | 28.97 ± 1.61 | (10.58) |  | 28.41 ± 0.35 | (9.80) |  | 33.28 ± 1.26 | (10.56) |  | 38.16 ± 3.91 | (9.46) |
|  | Cysteine |  | 0 | (0.00) |  | 0 | (0.00) |  | 3.34 ± 0.15 | (1.15) |  | 0.37 ± 0.23 | (0.12) |  | 0.00 | (0.00) |
|  | **Glutamic acid** |  | 29.65 ± 5.59 | (11.73) |  | 35.8 ± 2.22 | (13.07) |  | 33.63 ± 0.65 | (11.60) |  | 43.01 ± 1.82 | (13.65) |  | 20.56 ± 4.3 | (5.10) |
|  | Glycine |  | 11.11 ± 2.39 | (4.40) |  | 12.41 ± 0.92 | (4.53) |  | 12.63 ± 0.12 | (4.36) |  | 13.48 ± 0.47 | (4.27) |  | 57.59 ± 6.64 | (14.28) |
|  | **Proline** |  | 11.18 ± 2.9 | (4.42) |  | 16.25 ± 1.04 | (5.93) |  | 22.89 ± 0.3 | (7.89) |  | 27.65 ± 2.37 | (8.77) |  | 40.82 ± 3.17 | (10.12) |
|  | Serine |  | 14.68 ± 2.88 | (5.81) |  | 16.39 ± 1.14 | (5.98) |  | 16.22 ± 0.08 | (5.59) |  | 17.02 ± 0.39 | (5.40) |  | 19.75 ± 3.89 | (4.90) |
|  | Tyrosine |  | 11.93 ± 2.67 | (4.72) |  | 13.1 ± 1.55 | (4.78) |  | 12.44 ± 0.06 | (4.29) |  | 12.84 ± 0.47 | (4.07) |  | 30.7 ± 2.36 | (7.61) |
|  |  |  |  |  |  |  |  |  |  |  |  |  |  |  |  |  |
| Total concentration (mg/g) | |  | 252.85 ± 52.91 | |  | 273.94 ± 19.81 | |  | 290 ± 2.52 | |  | 315.23 ± 10.53 | |  | 403.39 ± 29.47 | |

**Supplementary Table S2.** Amino acid composition from the pollen loads of invasive plants as well as native plants. The concentrations are expressed in mg/g (mean ± sd) and as percentage of total amino acids in brackets, except for *L. salicaria* (mean).

|  |  |  |  |  |  |  |  |  |  |  |  |  |  |  | |  | |  |  |
| --- | --- | --- | --- | --- | --- | --- | --- | --- | --- | --- | --- | --- | --- | --- | --- | --- | --- | --- | --- |
|  |  |  | **Invasive plants** | | | | |  | **Native plants** | | | | | | | | | | |
|  |  |  | *Impatiens glandulifera* (n=3) | |  | *Buddleia davidii* (n=3) | |  | *Calluna vulgaris* (n=3) | |  | *Lythrum salicaria* (n=2) | | |  | | *Trifolium pratense* (n=3) | | |
|  |  |  |  |  |  |  |  |  |  |  |  |  |  |  | |  | |  |  |
| Essential amino acids | Arginine |  | 7.32 ± 1.5 | (4.90) |  | 9.34 ± 1.11 | (6.16) |  | 15.15 ± 0.23 | (7.83) |  | 8.91 | (6.19) |  | | 9.62 ± 1.56 | | (5.22) |  |
|  | Histidine |  | 7.99 ± 0.14 | (5.35) |  | 5.26 ± 0.69 | (3.47) |  | 6.04 ± 0.06 | (3.12) |  | 4.86 | (3.37) |  | | 5.89 ± 0.96 | | (3.19) |  |
|  | Isoleucine |  | 8.09 ± 0.2 | (5.42) |  | 7.67 ± 0.89 | (5.05) |  | 8.98 ± 0.08 | (4.64) |  | 6.52 | (4.53) |  | | 8.79 ± 1.43 | | (4.76) |  |
|  | Leucine |  | 12.5 ± 0.26 | (8.37) |  | 12.57 ± 1.29 | (8.29) |  | 14.62 ± 0.09 | (7.56) |  | 11.48 | (7.97) |  | | 13.9 ± 1.98 | | (7.53) |  |
|  | Lysine |  | 12.98 ± 0.36 | (8.69) |  | 13.68 ± 1.36 | (9.02) |  | 14.62 ± 0.19 | (7.56) |  | 12.17 | (8.45) |  | | 14.51 ± 1.85 | | (7.86) |  |
|  | Methionine |  | 3.92 ± 0.64 | (2.62) |  | 3.03 ± 1.4 | (2.00) |  | 4.75 ± 0.14 | (2.46) |  | 2.39 | (1.66) |  | | 3.41 ± 1.61 | | (1.85) |  |
|  | Phenylalanine |  | 7.9 ± 0.28 | (5.29) |  | 7.44 ± 0.92 | (4.90) |  | 9.37 ± 0.13 | (4.85) |  | 7.6 | (5.28) |  | | 9.06 ± 1.51 | | (4.91) |  |
|  | Threonine |  | 8.4 ± 0.28 | (5.63) |  | 7.54 ± 0.85 | (4.97) |  | 8.67 ± 0.06 | (4.48) |  | 6.78 | (4.71) |  | | 8.81 ± 1.31 | | (4.77) |  |
|  | Valine |  | 9.61 ± 0.21 | (6.44) |  | 10.07 ± 0.94 | (6.64) |  | 12.78 ± 0.2 | (6.61) |  | 9.06 | (6.29) |  | | 10.65 ± 1.33 | | (5.77) |  |
|  |  |  |  |  |  |  |  |  |  |  |  |  |  |  | |  | |  |  |
|  | Alanine |  | 8.64 ± 0.16 | (5.79) |  | 7.6 ± 0.98 | (5.01) |  | 10.83 ± 0.13 | (5.60) |  | 7.1 | (4.93) |  | | 8.97 ± 1.22 | | (4.86) |  |
|  | **Aspartic acid** |  | 16.85 ± 0.58 | (11.28) |  | 18.13 ± 1.98 | (11.95) |  | 18.34 ± 0.25 | (9.49) |  | 14.93 | (10.36) |  | | 17.65 ± 1.98 | | (9.57) |  |
|  | Cysteine |  | 0.64 ± 0.16 | (0.00) |  | 0 | (0.00) |  | 0.38 ± 0.21 | (0.20) |  | 0 | (0.00) |  | | 0 | | (0.00) |  |
|  | **Glutamic acid** |  | 18.96 ± 0.66 | (12.70) |  | 19.68 ± 2.15 | (12.97) |  | 21.53 ± 0.17 | (11.13) |  | 18.85 | (13.09) |  | | 20.13 ± 2.58 | | (10.91) |  |
|  | Glycine |  | 6.32 ± 0.12 | (4.23) |  | 6.57 ± 0.75 | (4.33) |  | 9.49 ± 0.08 | (4.91) |  | 6.05 | (4.20) |  | | 7.14 ± 1.08 | | (3.87) |  |
|  | **Proline** |  | 6.24 ± 0.1 | (4.18) |  | 7.73 ± 1.18 | (5.09) |  | 20.11 ± 0.79 | (10.40) |  | 13.94 | (9.68) |  | | 28.97 ± 3.72 | | (15.70) |  |
|  | Serine |  | 8.94 ± 0.31 | (5.99) |  | 9.14 ± 0.88 | (6.02) |  | 10.68 ± 0.07 | (5.52) |  | 8.12 | (5.64) |  | | 9.58 ± 1.36 | | (5.19) |  |
|  | Tyrosine |  | 4.66 ± 2.3 | (3.12) |  | 6.27 ± 0.84 | (4.14) |  | 7.01 ± 0.04 | (3.63) |  | 5.26 | (3.65) |  | | 7.42 ± 1.27 | | (4.02) |  |
|  |  |  |  |  |  |  |  |  |  |  |  |  |  |  | |  | |  |  |
| Total concentration (mg/g) | |  | 149.31 ± 6.71 | |  | 151.72 ± 16.44 | |  | 193.33 ± 1.45 | |  | 144.03 ± 5.4 | |  | | 184.49 ± 26.6 | | |  |

**Supplementary Table S3.** Pollen foraging parameters.

|  |  |  |  |  |  |  |  |  |  |  |  |  |  |  |  |
| --- | --- | --- | --- | --- | --- | --- | --- | --- | --- | --- | --- | --- | --- | --- | --- |
|  |  | **Invasive plants** | | | | |  | **Native plants** | | | | | | | |
|  |  | *Impatiens glandulifera* | |  | *Buddleia davidii* | |  | *Calluna vulgaris* | |  | *Lythrum salicaria* | |  | *Trifolium pratense* | |
| Visiting rate (number of visits/min) |  | 3.81 ± 0.81 (n = 22) | |  | 29.53 ± 6.71 (n = 16) | |  | 20.33 ± 3.96 (n = 15) | |  | 12.54 ± 3.41 (n = 8) | |  | 2.67 ± 0.57 (n = 11) | |
|  |  |  |  |  |  |  |  |  |  |  |  |  |  |  |  |
| Foraging time (min) |  | 30.37 ± 16.78 (n = 18) | |  | 30.88 ± 15.27 (n = 41) | |  | 33.17 ± 13.05 (n = 20) | |  | 36.32 ± 24.20 (n = 11) | |  | 54.70 ± 19.40 (n = 14) | |
|  |  |  |  |  |  |  |  |  |  |  |  |  |  |  |  |
| Number of foraging trips |  | 19 | |  | 42 | |  | 22 | |  | 12 | |  | 15 | |
|  |  |  |  |  |  |  |  |  |  |  |  |  |  |  |  |
| Surface of pollen loads (cm²) |  | 0.035 ± 0.014 (n = 19) | |  | 0.03 ± 0.009 (n = 42) | |  | 0.031 ± 0.016 (n = 22) | |  | 0.047 ± 0.008 (n = 12) | |  | 0.05 ± 0.013 (n = 15) | |
|  |  |  |  |  |  |  |  |  |  |  |  |  |  |  |  |
| Weight of pollen loads (mg) |  | 4.47 ± 1.82 (n = 19) | |  | 3.67 ± 1.19 (n = 42) | |  | 3.24 ± 1.67 (n = 22) | |  | 5.73 ± 1.03 (n = 12) | |  | 10.28 ± 2.82 (n = 15) | |
|  |  |  |  |  |  |  |  |  |  |  |  |  |  |  |  |
| Pollen intake (mg/h) |  | 11.26 ± 8.01 (n = 18) | |  | 8.47 ± 3.80 (n = 41) | |  | 6.29 ± 2.86 (n = 20) | |  | 13.89 ± 8.58 (n = 11) | |  | 13.12 ± 6.59 (n = 14) | |
|  |  |  |  |  |  |  |  |  |  |  |  |  |  |  |  |
| Amino acid intake (mg TAA/h) |  | 1.68 ± 1.19 (n = 18) | |  | 1.29 ± 0.57 (n = 41) | |  | 1.22 ± 0.55 (n = 20) | |  | 2 ± 1.24 (n = 11) | |  | 2.42 ± 1.21 (n = 14) | |
|  |  |  |  |  |  |  |  |  |  |  |  |  |  |  |  |
